# Anatomical study of perigastric fascial space and guidance for laparoscopic approach

**DOI:** 10.3389/fsurg.2024.1322079

**Published:** 2024-11-20

**Authors:** Guoliang Jin, Shuquan Duan, Ana Guan, Zhepeng Wang, Ranhao Zhang, Wenjuan Qiao, Qiuhong Wang, Liansheng Zheng

**Affiliations:** ^1^ Graduate School, Baotou Medical College, Baotou, Inner Mongolia, China; ^2^ Digestive Department, The Second Affiliated Hospital of Baotou Medical College, Baotou, Inner Mongolia, China

**Keywords:** stomach cancer, fusion fascia, fascial space, laparoscopes, surgery

## Abstract

**Objective:**

To study the anatomic characteristics of gastric peritoneum fascia space and provide a safe surgical approach for laparoscopic radical gastrectomy.

**Method:**

The morphological characteristics of perigastric fascia and fascial space and the course of important blood vessels were observed and studied in 2 fresh adult cadavers, 5 formalin immersed cadavers and 56 patients undergoing total gastrectomy. The hemoglobin, albumin, prealbumin, total protein, operation time, intraoperative blood loss, number of lymph node dissection and the incidence of complications before and after laparoscopic total gastrectomy with different approaches were statistically analyzed.

**Results:**

Through dissecting the cadaver, it is found that the space of the posterior gastric fascia space is suitable for laparotomy and laparoscopic surgery. The space between the prepancreatic fascia and the posterior gastric fascia is located in a plane, and the posterior gastric fascial space is connected with the gastrosplenic space and hepatogastric space. Through three different plane approaches, all can enter the space without blood vessels and nerves, so as to achieve complete gastrectomy. Statistical analysis of patients undergoing total gastrectomy with different approaches showed that there was no significant difference in operation time, intraoperative blood loss, number of lymph node dissection and postoperative complications among the three approaches (*P* > 0.05). There was no significant difference in postoperative hemoglobin, albumin, prealbumin and total protein (*P* > 0.05).

**Conclusions:**

The space of the posterior gastric fascia space is suitable for laparotomy and laparoscopic surgery**.**the application of the anatomical study of perigastric fascial space in laparoscopic radical resection of gastric cancer not only accords with the tumor-free principle of radical resection of tumor, improves the safety of operation, but also reduces the occurrence of complications such as bleeding and important organ injury.

## Highlights

• Space of the posterior gastric fascia space is suitable for laparotomy and laparoscopic surgery.
• Surgical removal of tumors in different positions can be executed through the gastrohepatic space, gastrosplenic space, and gastrocolon space.

## 1 Introduction

Gastric cancer is one of the most common malignant tumors of digestive tract in China. The early symptoms of gastric cancer are atypical and often reach the advanced stage when they are found (1–3). Surgery is still the main treatment for gastric cancer, with the rapid development of laparoscopic surgery, the research focus of retroperitoneal space has gradually shifted to the field of embryological space (fusion fascia space) between gastrointestinal mesentery and abdominal wall. The theory of perirectal fascia and space is relatively mature, while the structure of perigastric and peripancreatic fascial space has not been thoroughly elucidated. Because of the multiple mesentery around the stomach, pancreas and duodenum, the anatomy of these mesenteries and their surrounding spaces is the theoretical basis for the operation of gastric cancer (4). With the rise of laparoscopic surgery for gastric cancer, perigastric, peripancreatic and peri-duodenal fascia spaces have gradually become the research focus, at present, most studies are based on laparoscopic surgery or imaging observation. Some studies have proposed the anatomical study of the perigastric fascial space centered on the pancreas. There are many different surgical methods and approaches for laparoscopic total gastrectomy (5, 6). We observed the perigastric and peripancreatic fascia spaces and blood vessels through autopsy to guide the surgical approach of laparoscopic total gastrectomy.

## 2 Materials and methods

### 2.1 Materials

#### 2.1.1 Research objects

There are 2 fresh corpses, 5 formalin soaked corpses, a set of Olympus laparoscopes and an Olympus video recorder.

Fifty-six patients who underwent laparoscopic total gastrectomy in Second Affiliated Hospital of Baotou Medical College from September 2018 to September 2021 were selected. According to different surgical approaches, the patients were divided into group A (*n* = 17), group B (*n* = 21) and group C (*n* = 18). The surgical approach in group A was medial approach, group B was caudal approach, and group C was caudal combined lateral approach. Informed consent was obtained from each of the participants. The age and male and female composition of the three groups are shown in Table 1, and the clinicopathological data of the patients are shown in Table 2.

**Table 1:** Basic information of the patient.

Groups	Cases (*n*)	Male/female	Body mass index(BMI)	Age (years)
A	17	12/5	22.23 ± 2.80	63.94 ± 7.66
B	21	18/3	22.56 ± 2.86^a^	69.05 ± 6.70^a^
C	18	13/5	22.73 ± 4.18^a,b^	66.11 ± 7.92^a,b^
*F*			0.099	2.072
*p*			0.906	0.136

^a^ compare to the group A, *P* > 0.05

^b^ compare to the group B, *P* > 0.05.

**Table 2:** Clinicopathological data of patient cases.

Clinicopathological factors	Cases	*P* %
Gender		
Male	43	76.79
Female	13	23.21
Tumor site		
medial approach	17	30.36
caudal approach	21	37.50
caudal combined lateral approach	18	32.14
Degree of differentiation		
Poor differentiation	18	32.14
Medium-low differentiation	17	30.36
Middle differentiation	13	23.21
Well-medium differentiation	2	3.57
Others	6	10.72
Tumor size		
<5	28	50
≥5	28	50
Regional lymph node metastasis		
N0	17	30.36
N1	30	53.57
N2	8	14.28
N3	1	1.79
Infiltrating depth		
T1	7	12.50
T2	3	5.36
T3	4	7.14
T4	42	75

#### 2.1.2 Inclusion and exclusion criteria

Inclusion criteria: I. All patients were diagnosed with gastric cancer by endoscopy and pathology, II. All patients were able to tolerate surgical treatment, III. There was no distant metastasis in preoperative imaging examination, IV. The patient did not receive radiotherapy and chemotherapy before operation. All the patients agreed to the operation plan and could cooperate with this study; VI. All operations were performed by laparoscopy.

Exclusion criteria: I. Cardiopulmonary and renal function disease, poor systemic nutrition; II. Emergency operation with obstruction and bleeding; III. The patient had a history of abdominal surgery, and IV The patients who had distant metastasis after imaging examination.

#### 2.1.3 Observation index

The postoperative hospitalization time, hospitalization cost, operation time, intraoperative blood loss, number of postoperative lymph node dissection, preoperative hemoglobin, preoperative albumin, preoperative total protein, hemoglobin on the 3rd day after operation, prealbumin, total protein and the incidence of postoperative complications were compared among the three groups.

### 2.2 Methods

The morphological characteristics of perigastric fascial space and the distribution of perigastric vessels were observed on cadaveric specimens, and the distribution of perigastric mesentery and the course of blood vessels were observed from the perspective of laparoscopy.The anatomical morphology was recorded by photos and videotape. Combining with the characteristics of embryonic dorsal mesentery to guide the safe surgical approach and plane of laparoscopic total gastrectomy.

#### 2.2.1 Autopsy

**Figure 1:**
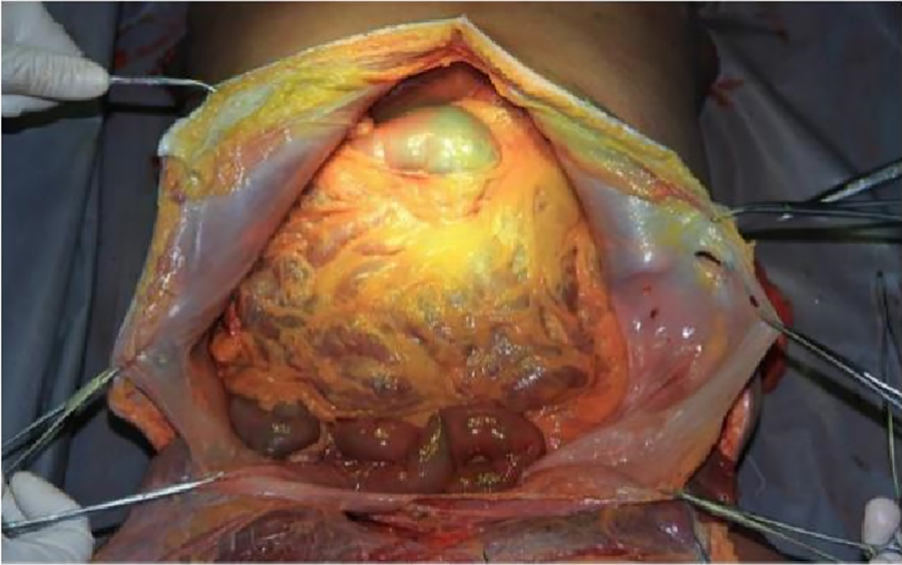
Full expose of the abdominal cavity to the abdominal organs. PHA, proper hepatic artery; GDA, gastroduodenal artery; CHA, common hepatic artery; Sa, spleen artery; CA, celiac axis; LGA, left gastric artery; RGS, retrofascial gastric space; GPL, gastrophrenic ligament.

**Figure 2:**
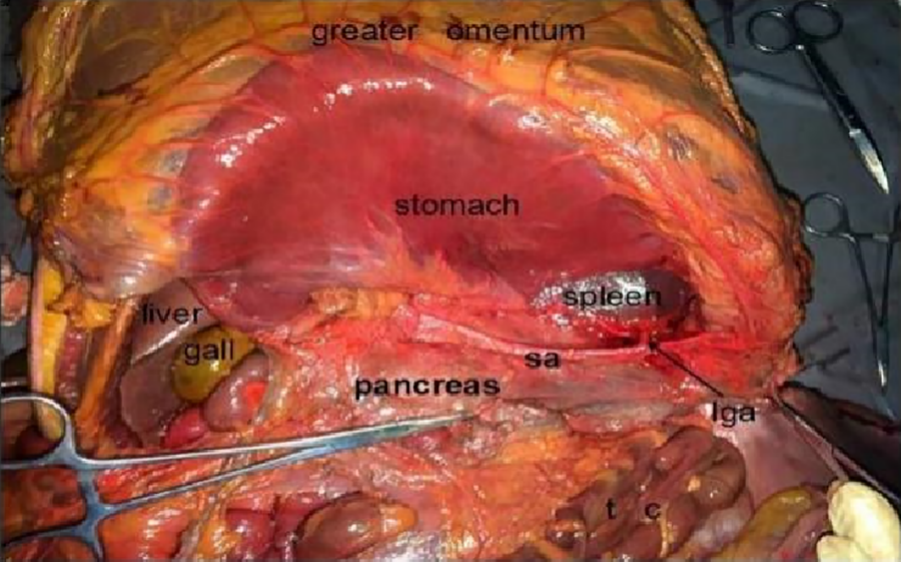
Prepancreatic fascia space. PHA, proper hepatic artery; GDA, gastroduodenal artery; CHA, common hepatic artery; Sa, spleen artery; CA, celiac axis; LGA, left gastric artery; RGS, retrofascial gastric space; GPL, gastrophrenic ligament.

**Figure 3:**
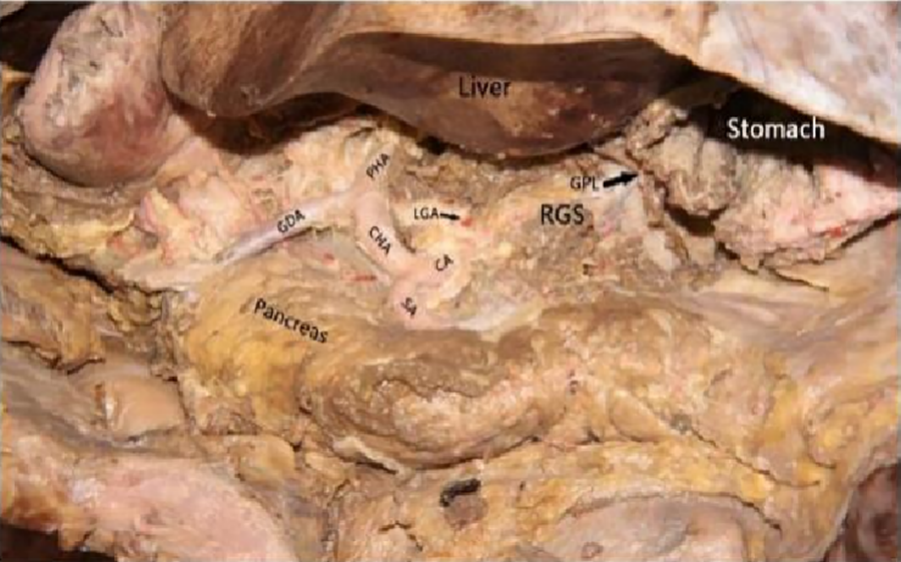
Celiac axis and other major vascular branches go. PHA, proper hepatic artery; GDA, gastroduodenal artery; CHA, common hepatic artery; Sa, spleen artery; CA, celiac axis; LGA, left gastric artery; RGS, retrofascial gastric space; GPL, gastrophrenic ligament.

The cadaver was placed on the autopsy table, making a longitudinal incision about 35 cm in the middle of the abdomen, cutting the skin and subcutaneous tissue in turn, and fully exposing the organs of the abdominal cavity (Figure 1). According to the surgical path of total gastrectomy, pulling the stomach and the greater omentum to the head, the mesenteric band connecting the transverse colon and the transverse colon can be observed. Incising the anterior lobe of the transverse mesocolon, entering the transverse mesocolon space, and continuing to dissociate the anterior lobe of the transverse colon to the root of the mesenter. at this time, it can be seen that the two layers of the mesentery root are fused at the lower edge of the pancreas, and are divided into two layers, from the inferior margin of the pancreas forward, stripping the capsule of the pancreas, it can be seen that there is a loose fascial space without blood vessels and nerves between the pancreatic capsule and the pancreatic parenchyma, that is, the anterior pancreatic space. It can be observed that there is a large space in the anterior pancreatic space, which is surrounded by stomach, transverse colon, liver, spleen and pancreas (Figure 2), when the pancreatic capsule is cut to the upper edge of the pancreas, the common hepatic artery is separated from the celiac trunk to the right, and the common hepatic artery is separated from the gastroduodenal artery at the junction of stomach and pancreas. The common hepatic artery divides into two proper hepatic arteries into the liver after the hepatoduodenal ligament divides into the right gastric artery. The celiac trunk divides the splenic artery to the left and downward, and the splenic artery runs along the upper edge of the pancreas, divides several blood vessels along the way to supply the pancreas (Figure 3), and separates two splenic lobar arteries near the splenic hilum into the spleen. The celiac trunk divides the left gastric artery upward, and the left gastric artery is often severed here during distal radical gastrectomy or total gastrectomy, which is an important anatomical sign of gastrectomy.

Turning the lower edge of the pancreas to the side of the head, Peeling downward and backward from the inferior edge of the pancreas, the splenic vein is located at the back of the body and tail of the pancreas and receives reflux from the inferior mesenteric vein. Continuing to peel off the body and tail of the pancreas along the fascial space to the caudal side of the pancreas. there is a loose non-vascularized fascial space (Toldt fascia space) between the posterior part of the body and tail of the pancreas and the prerenal fascia, Toldt fascia space is connected to the posterior gastric fascia space upward, and there are no blood vessels and nerves in this space. The splenic flexure of the colon and the descending colon were dissociated medially along the posterior colonic fascial space. It was found that the Toldt fascial space was located in a plane with the Toldt fascial space behind the rectum, and there were no blood vessels and nerves in this space.

Turning the left lobe of the liver to the upper right, it can be seen the hepatogastric ligament, which contains the right gastric vessels and accessory hepatic arteries. The hepatogastric ligament is cut into the hepatogastric space, and the hepatogastric space is connected to the lower segment of the esophagus to the head, and the gastrophrenic ligament is cut open to turn the lesser curvature of the stomach to the left. it can be seen that the hepatogastric space is connected with the retrogastric space, and there are no blood vessels and nerves in this space, therefore, NO. 1, 3, 5 and 12 groups of lymph nodes can be dissected in this space, and the amputation of duodenum can be completed in this space.

Turning the great curvature of the stomach to the upper right and incising the gastropancreatic ligament, the left gastroepiploic artery separated from the splenic artery can be seen. The left gastroepiploic artery is not only an important anatomical marker for radical resection of gastric cancer, but also an important marker for lymph node dissection in NO.4 group. After cutting off the left gastroepiploic artery, we can enter the gastrosplenic space, which is filled by short gastric vessels and lymphoid connective tissue, which can communicate with the left side of the esophagus and medially with the posterior gastric fascia space, and in this space, lymph node dissection in groups NO2, 4 and 10 can be cue completely.

Through autopsy, it can be found that there is a large space between the posterior gastric fascia, which is often used for the operation of stomach, spleen, pancreas and colon. This space communicates with the gastrosplenic space to the left and fuses with the hepatogastric space to the right, and there are no blood vessels and nerves in this space, which lays an anatomical foundation for total gastrectomy.

#### 2.2.2 Laparoscopic surgery

According to the tumor-free principle of tumor resection, we should minimize the clamp and touch of the tumor during the operation, so as to reduce the spread of tumor during operation, recurrence and metastasis after operation, and benefit the patients.

##### 2.2.2.1 Caudal approach

**Figure 4:**
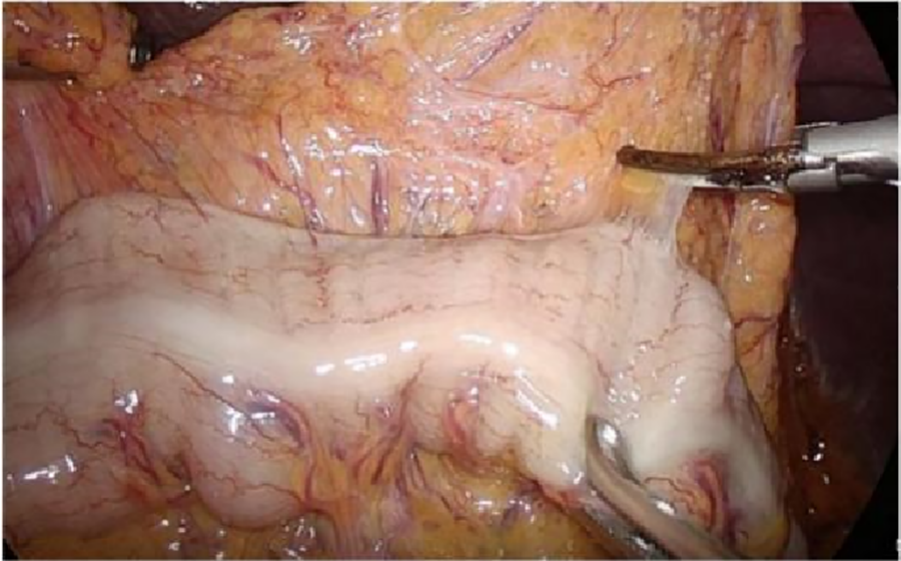
Laparoscopic incision of the aperitif colon ligament. PHA, proper hepatic artery; GDA, gastroduodenal artery; CHA, common hepatic artery; Sa, spleen artery; LGA, left gastric artery; GCV, gastric coronary vein.

**Figure 5:**
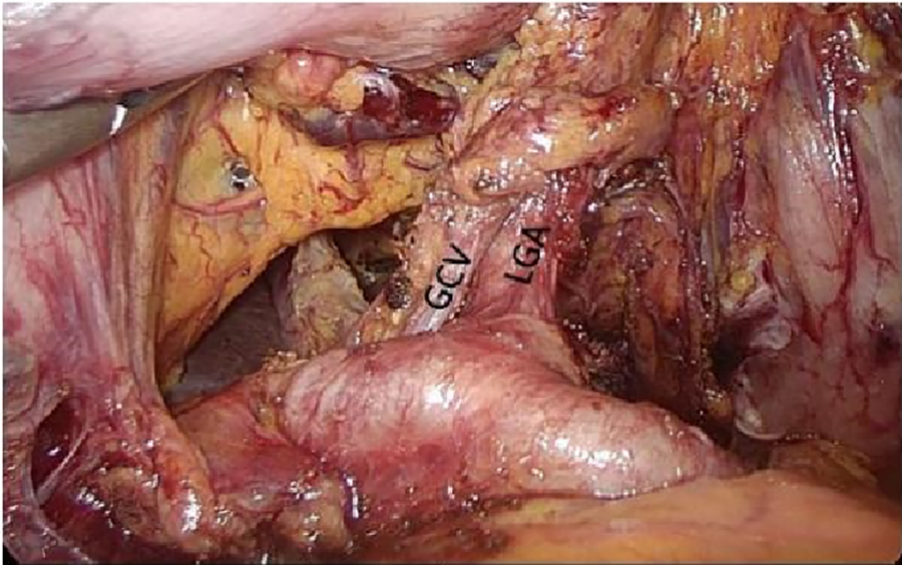
Left gastric artery and gastric coronary vein. PHA, proper hepatic artery; GDA, gastroduodenal artery; CHA, common hepatic artery; Sa, spleen artery; LGA, left gastric artery; GCV, gastric coronary vein.

**Figure 6:**
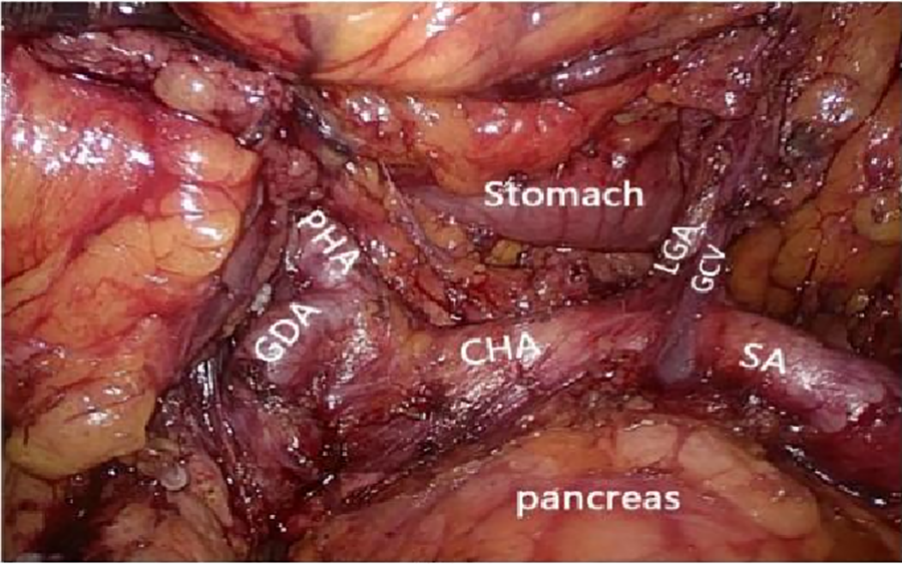
Celiac axis and other major vascular branches go. PHA, proper hepatic artery; GDA, gastroduodenal artery; CHA, common hepatic artery; Sa, spleen artery; LGA, left gastric artery; GCV, gastric coronary vein.

After laparoscopic exploration, the greater omentum was pulled to the cephalic side and expanded to both sides, while the transverse colon was pulled downward, close to the free omentum in the vascularized area of the upper edge of the transverse colon (Figure 4), and entered the posterior gastric fascia space. Dissociating the greater omentum to the left to the inferior spleen and to the right to the liver region of the colon. The antrum of the stomach was drawn to the head, and the anterior lobe of the transverse mesocolon was sharply dissected with an ultrasonic knife to the medial edge of the duodenum, and the omentum and transverse mesocolon were dissociated to the pylorus along the medial margin of the duodenum, where the superior anterior pancreaticoduodenal vein could be seen. The right gastroomental vein was dissociated to the upper edge of pancreas, and the lymph nodes of group No. 6 were dissected, the right gastroepiploic vein was cut off at the superior anterior pancreaticoduodenal vein, right colonic vein and above the confluence of Henle's trunk. Free the end of the gastroduodenal artery and the root of the right gastroepiploic artery, cut off the right gastroepiploic artery at the root of it and separate the medial wall of the duodenum along the gastroduodenal artery. Pulling the gastric body upward and forward, exposing the upper margin of the pancreas, removing the No. 8a group lymph nodes to the right along the front of the common hepatic artery, exposing the initial segment of the splenic artery, and dissecting the proximal splenic artery lymph nodes of the No. 11p group along the surface of the superior margin of the pancreas. Expanding the Toldt space, exposing the diagram pars, dissociating the gastric wall of the lesser curvature of the gastric body, removing the lymph nodes in the No. 9 group, and exposing the gastric coronary vein and lefting gastric artery (Figures 5, 6), the coronary vein was severed at the upper edge of the common hepatic artery, the lymph nodes in the No. 7 group were removed, the left gastric artery was cut off, and the marginal lymph nodes on the right side of the celiac artery were cleared. Incising the gastrophrenic ligament, dissociating upward to the right edge of the esophagus, removing the lymph nodes of No. 8a group along the back of the common hepatic artery, exposing the portal vein, and removing the lymph nodes of No. 12p group.

**Figure 7:**
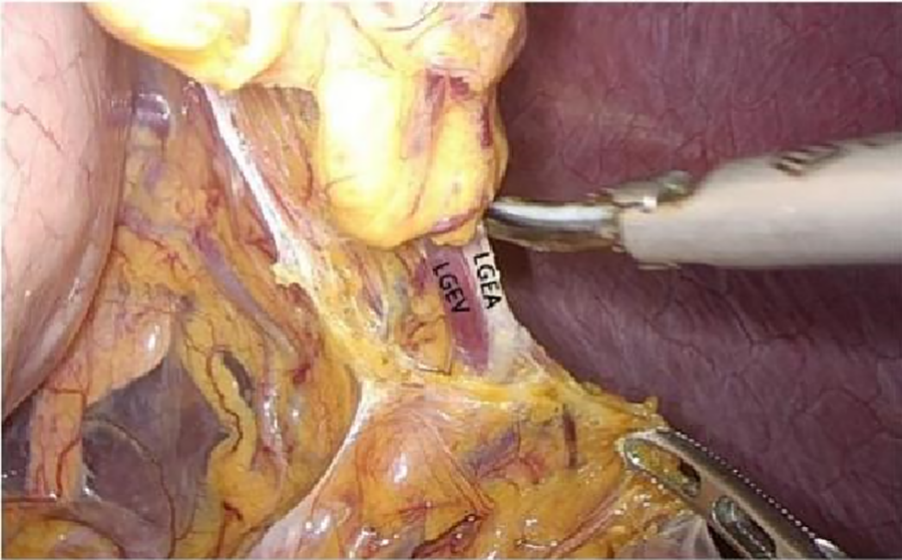
Open the appetizer splenic ligament from the left into the posterior gastric fascia space. LGEA, left gastroepiploic artery; LGEV, left gastroepiploic vein; GHL, gastrohepatic ligament; DP, diagram pars; RGS, retrofacial gastric space.

Turning the gastric body to the upper right, exposing the lower pole of the spleen, skeletonizing the end of the main splenic vessel along the upper edge of the tail of the pancreas, then gradually exposing the left gastroepiploic vessels (Figure 7), sweepting up the lymph nodes in the No.4sb group, preserving the lower pole of the splenic vessels, cutting off the root of the left gastroepiploic vessels, skeletalizing the splenic artery along the direction of the splenic artery to the splenic hilum. Sweeping up the lymph nodes of the No.11d group, exposing the upper pole area of the spleen and completing the splenic hilar lymph node dissection. Cutting open the gastrosplenic ligament, cutting off and ligate the short gastric vessels, and entering the posterior gastric fascia space from the gastrosplenic space.

The duodenum was cut off and closed with Endo-GIA Stapler, the root of the right gastric vessel was skeletalized along the direction of the proper hepatic vessels, and the lymph nodes in the No.5 group were swept up. The No.12a lymph nodes on the surface of the proper hepatic vessels were cleared. The hepatogastric ligament was severed along the inferior edge of the liver until the right edge of the esophagus, the right and posterior lymph nodes of the esophagus were cleared, the esophagus was skeletalized, and the esophagus was cut off in the lower part of the esophagus. So far, the lower part of the esophagus, the whole stomach and duodenal bulb, as well as the attached omentum and lymph nodes have been completely removed.

##### 2.2.2.2 Medial approach

**Figure 8:**
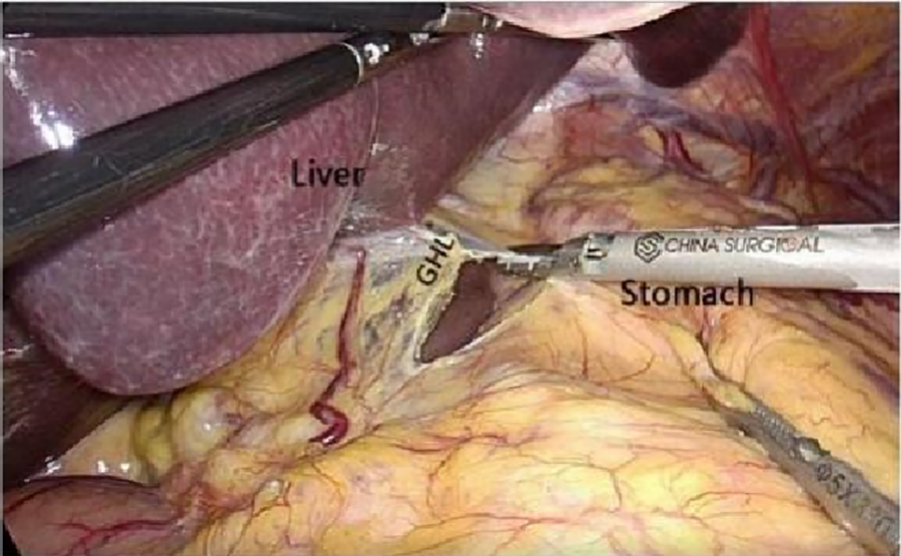
Laparoscopic incision of the hepatogastric ligament. LGEA, left gastroepiploic artery; LGEV, left gastroepiploic vein; GHL, gastrohepatic ligament; DP, diagram pars; RGS, retrofacial gastric space.

**Figure 9:**
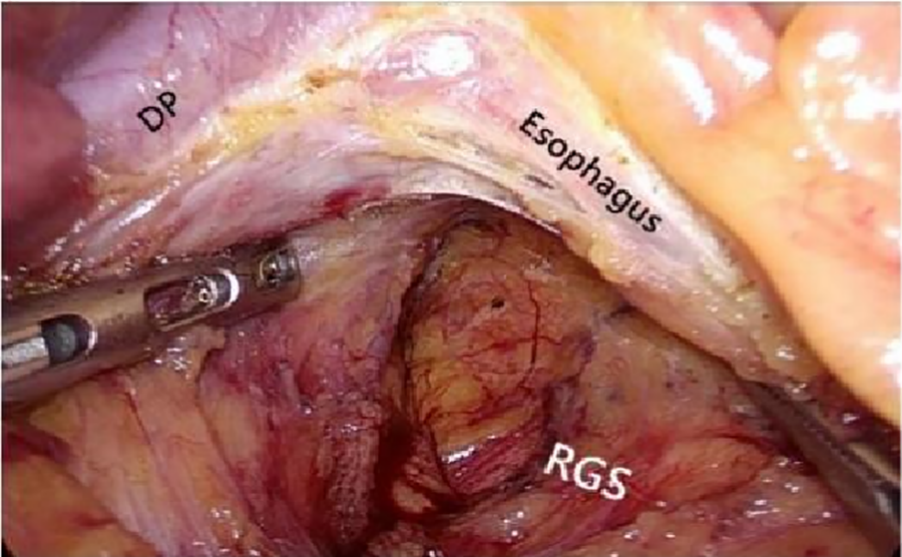
The hepatogastric ligament is opened laparoscopically and entered from the right side into the Retrofascial gastric space. LGEA, left gastroepiploic artery; LGEV, left gastroepiploic vein; GHL, gastrohepatic ligament; DP, diagram pars; RGS, retrofacial gastric space.

After laparoscopic exploration, cutting the hepatogastric ligament (Figure 8). The gastrophrenic ligament was cut between the stomach and the the right diagram pars into the retrofascial gastric fascia space (Figure 9), the retrofascial gastric fascia space was loose without blood vessels and nerves, and extended upward to the posterior esophagus and downward to the superior border of the pancreas, No.1, group 3 and group 5 lymph nodes were cleared and endoscopic gauze was placed. On the one hand, endoscopic gauze can oppress and stop bleeding for small bleeding, on the other hand, it can guide the caudal approach.

Cutting open the gastrocolic ligament and dissociate the transverse mesocolon to the superior border of the pancreas, and entering the posterior gastric fascia space under the guidance of endoscopic gauze. sweeping up the lymph nodes of groups No. 6, 7, 8, 9, 11 and 12, using Endo-GIA Stapler to cut off and close the duodenum. The distal part of the stomach was pulled up to the right, and the lymph nodes of No.4, 10 and 2 groups were swept up. Resection of lower esophagus, total stomach, duodenal bulb, attached omentum and lymph nodes were completed.

##### 2.2.2.3 Caudal combined lateral approach

After the completion of endoscopic exploration, incising the gastrocolic ligamenta and dissociating the transverse mesocolon to the superior border of the pancreas, sweeping up the lymph nodes in groups No. 6, 7, 8 and 9. Entering the Retrofascial Gastric Space and expand the space to the liver and spleen. Then along the splenic artery to the greater curvature of the stomach, the left gastroepiploic vessel was cut off, and the lymph nodes in No. 4sb group were swept up. Cutting open the gastrosplenic ligament, and connecting the gastrosplenic space with the retrofascial gastric space, and dissociate upward to the left side of the esophagus. Cutting off the duodenum and right gastric vessel, sweeping up No. 12, 5 groups of lymph nodes. Cutting open the hepatogastric ligament, entering the retrofascial gastric space, and extending upward to the right side of the esophagus. At this point, the resection of the lower esophagus, the whole stomach and duodenal bulb and the attached omentum and lymph nodes were completed.

#### 2.2.3 Laparoscopic surgery statistical processing analysis

The data were analyzed by SPSS22.0 statistical software, and the counting data were expressed by the number of cases (%), compared with ×2 test, and the measurement data were expressed by statistical ± s, using F test/*t*-test. The difference was statistically significant (*p* < 0.05).

## 3 Result

**Figure 10:**
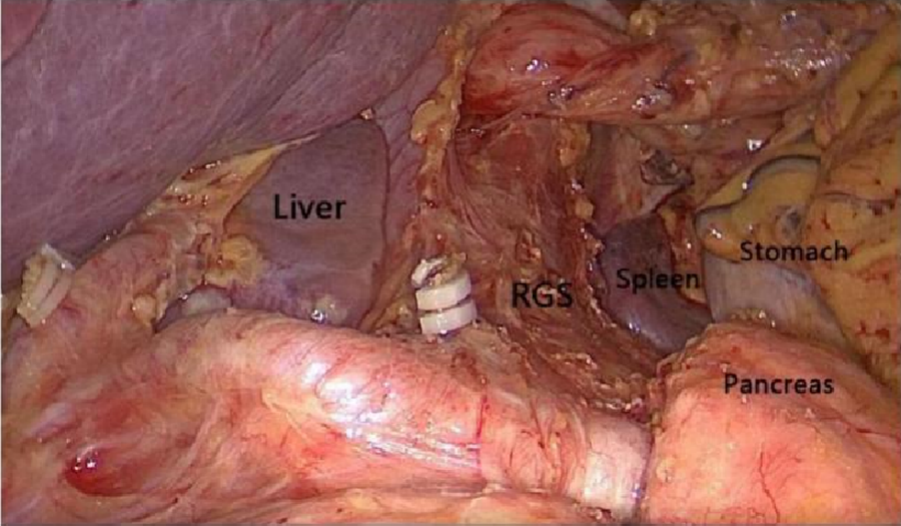
The space between the prepancreatic fascia space and the retrofascial gastric space is located in a plane, the retrofascial gastric space communicates with the hepatogastric space to the right and communicates with the gastrosplenic space to the left.

Through the autopsy of the perigastric and peripancreatic fascial space, we confirmed that the space of the retrofascial gastric space is large, which is suitable for laparotomy and laparoscopy. The retrofascial gastric space is a loose fascial space without blood vessels and nerves. The retrofascial gastric space and the prepancreatic fascia space are located in a plane, which communicates with the hepatogastric space to the right and to the gastrosplenic space to the left (Figure 10). When performing total gastrectomy, we can enter the retrogastric fascia space by incising the prepancreatic fascia space, or incising the hepatogastric ligament into the hepatogastric space to communicate with the retrogastric fascia space, and also incising the gastrosplenic ligament into the gastrosplenic space to communicate with the retrogastric fascia space. Therefore, there are three different surgical approaches, which can enter the posterior gastric fascia space, so as to achieve complete gastrectomy.

There was no significant difference in hemoglobin, albumin, prealbumin and total protein among the three groups of patients with different approaches before operation (*p* > 0.05). There was no significant difference in hemoglobin, albumin, prealbumin and total protein on the third day after operation (*p* > 0.05), as shown in Table 3.

**Table 3:** Comparison of preoperative and postoperative nutritional indicators of the three groups of patients with different surgical approaches.

Group	Hb (g/L)		ALB (g/L)		PA (mg/L)		TP (g/L)	
	Before operation	3rd after operation	Before operation	3rd after operation	Before operation	3rd after operation	Before operation	3rd after operation
A	129.65 ± 24.97	120.24 ± 23.58	39.40 ± 3.61	33.09 ± 2.77	214.11 ± 48.04	116.71 ± 30.30	64.90 ± 10.46	58.11 ± 6.42
B	139.10 ± 19.24^a^	121.48 ± 17.18^a^	40.90 ± 5.59^a^	35.46 ± 4.58^a^	205.01 ± 12.06^a^	106.54 ± 27.53^a^	67.15 ± 7.14^a^	57.30 ± 5.83^a^
C	128.56 ± 20.06^a,b^	114.83 ± 18.94^a,b^	39.03 ± 4.68^a,b^	33.04 ± 4.25^a,b^	210.03 ± 54.32^a,b^	102.86 ± 33.56^a,b^	64.72 ± 8.56^a,b^	55.23 ± 7.01^a,b^
F Value	1.442	0.643	0.884	2.352	0.141	0.973	0.485	0.952
*P* Value	0.246	0.530	0.419	0.105	0.869	0.384	0.618	0.392

^a^ compare to the group A, *P* > 0.05

^b^ compare to the group B, *P* > 0.05.

The operation time, blood loss, lymph node dissection, postoperative hospitalization time and hospitalization cost of the patients with different approaches in the three groups were roughly the same (*p* > 0.05), as shown in Table 4.

**Table 4:** Comparison of operation-related data in three groups of patients with different surgical approaches.

Group	Operation time (min)	Amount of bleeding (ml)	Number of lymph node sweeps (n)	Postoperative hospital stay (d)	Cost of hospitalization (CNY)
A group	261.29 ± 81.70	50.00 (20.00,100.00)	44.76 ± 16.57	14.88 ± 7.44	68,742.24 ± 17,416.36
B group	272.10 ± 85.94^a^	50.00 (20.00,100.00)	35.86 ± 13.28^a^	14.71 ± 4.86^a^	68,199.84 ± 15,848.97^a^
C group	281.83 ± 73.14^a,b^	90.00 (45.00,100.00)	38.56 ± 11.00^a,b^	14.44 ± 4.57^a,b^	67,422.66 ± 15,207.92^a,b^
F/H value	0.283	0.706	2.033	0.027	0.265
*P* value	0.755	0.703	0.141	0.974	0.769

^a^ compare to the group A, *P* > 0.05

^b^ compare to the group B, *P* > 0.05.

The postoperative complications of the three groups were as follows: 1 case of anastomotic leakage and 1 case of postoperative emptying disturbance in group A, with a total complication rate of 11.76%. There was 1 case of anastomotic leakage in group B, and the total incidence of complications was 4.76% in group B. there were 2 cases of pulmonary infection in group C, and the total incidence of complications was 11.11%. Due to the lack of sample size, the accurate Fisher test showed that there was no significant difference in the incidence of total complications among the three groups.

## 4 Discussion

During the embryonic period, the dorsal mesentery of stomach evolved into gastrosplenic ligament, splenorenal ligament, pancreatic fascia, anterior lobe of transverse mesocolon and greater omentum (7–10). During the embryonic period, the dorsal gastric mesentery formed a widely connected mesentery and fascia fusion in the abdominal cavity, because the dorsal gastric mesentery evolved into different layers and different planes of fascia, so different levels of fascia formed a different plane of non-vascular fascial space (7, 8, 11). The existence of these fascial spaces without blood vessels and nerves provides us with a safe surgical approach and plane (9).

Through autopsy, we found that the anterior pancreatic fascial space and the retrofascial gastric space were located in a plane, which had no blood vessels and nerves, and the retrofascial gastric space extended to both sides, which communicated with the hepatogastric space on the right and the gastrosplenic space on the left. It provides a safe operation plane for our operation.

On the basis of autopsy, we completed 56 cases of laparoscopic total gastrectomy through different surgical approaches, and all the patients recovered well. The surgical approaches are summarized as follows: (1) caudal approach: cutting the gastrocolic ligament into the retrofascial gastric space, peeling the pancreatic capsule to the superior border of the pancreas, skeletalizing the splenic artery, the common hepatic artery and the left gastric artery, and dissecting the lymph nodes in groups No. 7, 8 and 9 at the same time. The gastroduodenal artery was found along the common hepatic artery, and the lymph nodes in No. 6 group were swept up, the right gastroepiploic vessels were cut off and the left gastric vessels were cut off, the posterior gastric fascia space was expanded to the left and right, and the lymph nodes of lesser curvature and greater curvature of stomach were swept up. (2) medial approach: cut the hepatogastric ligament, enter the retrogastric fascia space directly from the hepatogastric space, expand the space upward and downward, place hemostatic gauze as a guide, and then cut the gastrocolic ligament and enter the retrogastric fascia space from the caudal side. Until it penetrates with the above-mentioned space, and then complete lymph node dissection in each group. (3) caudal combined lateral approach: cutting the gastrocolic ligament into the posterior gastric fascia space, expanding the space to the liver and spleen, and extending to the spleen, until the left gastroepiploic artery separated from the splenic artery, cutting off the left gastroepiploic blood vessel, cutting open the gastrosplenic ligament, entering the posterior gastric fascia space from the left approach, and reaching the angle of diaphragm upward, so as to completing the dissection of lymph nodes in each group.

Based on the statistical analysis of 56 patients undergoing total gastrectomy with different approaches, it was found that on the basis of following the tumor-free principle and no-touch principle, there was no significant difference in operation time, intraoperative blood loss, number of lymph node dissection, total incidence of postoperative complications and hospitalization costs among the three different approaches, and there was no significant difference in postoperative hemoglobin, albumin, prealbumin and total protein, and this is exactly what the surgeon expects. Similar to the other studies (8, 11, 12), it shows that the three surgical approaches are feasible and safe as long as they are familiar with perigastric and peripancreatic fascial spaces and vascular anatomy.

**Figure 11:**
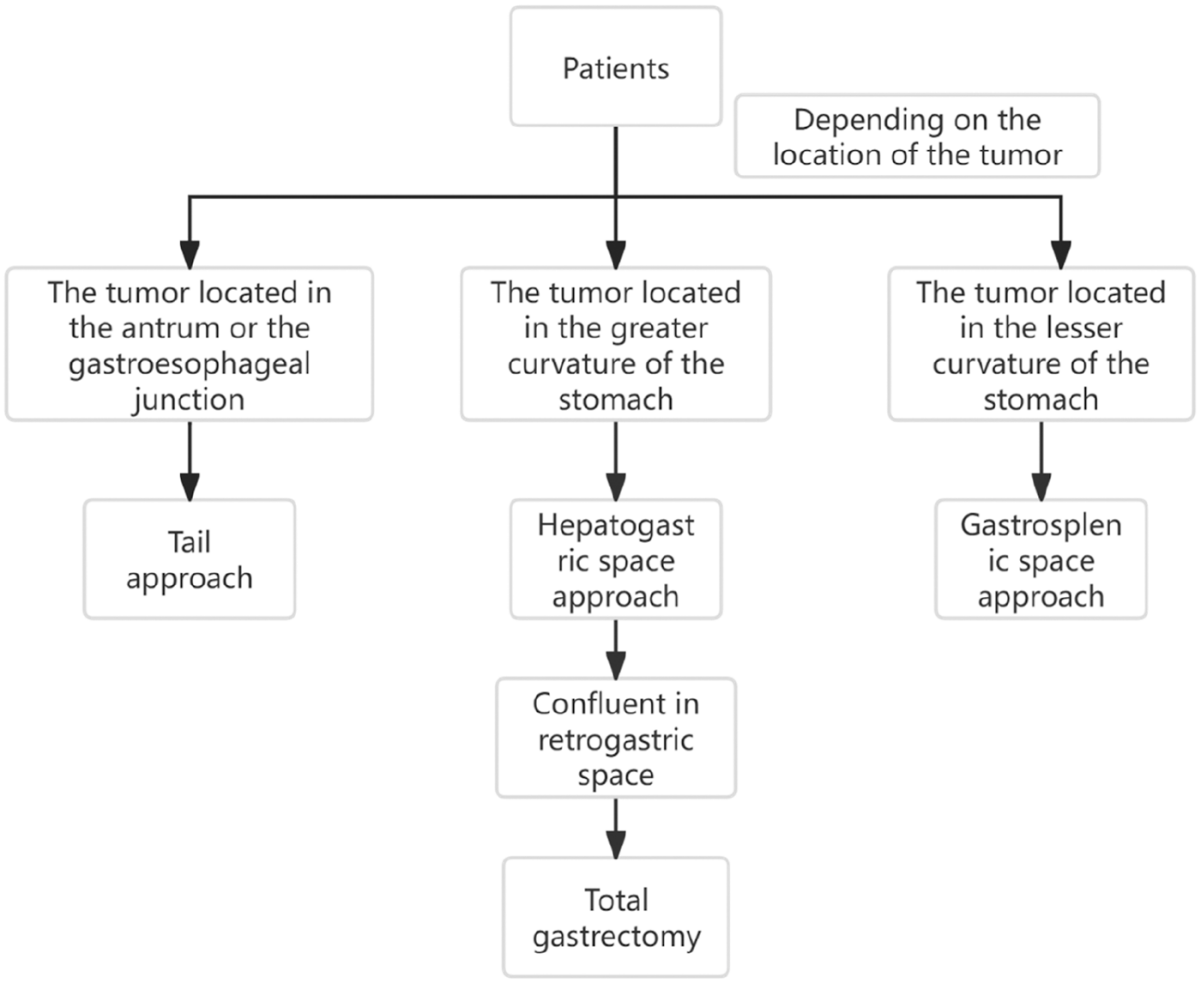
The schematic diagram of three surgical approaches for laparoscopic radical gastrectomy.

In summary, through the study, it is found that the fascial space around the stomach and pancreas is complex and connected with each other. The retrofascial gastric space and the prepancreatic fascial space are located in a plane, which communicates with the hepatogastric space to the right and the gastrosplenic space to the left. There are no blood vessels and nerves running in the retrofascial gastric space, which provides us with a safe operation plane. Only when we are familiar with these fascial spaces can we make a reasonable surgical approach according to the tumors in different locations. The anatomical study of perigastric fascial space can be effectively applied to laparoscopic radical resection of gastric cancer, which not only accords with the tumor-free principle and no-touch principle of radical resection of tumor, improves the safety of operation, but also reduces the occurrence of complications such as bleeding and injury of important organs (Figure 11). Limitations:Because the postoperative time of patients is short, most of them are within 1–2 years, so there is no difference in survival rate, recurrence rate and metastasis rate between the three groups. This study will continue, long-term follow-up and explore the long-term efficacy.

## Data Availability

The raw data supporting the conclusions of this article will be made available by the authors, without undue reservation.
